# Mixed methods assessment of an integrated hypertension and HIV care model: Acceptability, feasibility, and clinical outcomes at primary healthcare clinics in Wakiso District, Uganda

**DOI:** 10.4102/jphia.v17i1.1539

**Published:** 2026-01-13

**Authors:** Fred C. Semitala, John Baptist Kiggundu, Lilian Giibwa, Florence Ayebare, Isaac Ssinabulya, Jeremy I. Schwartz, Donna Spiegelman, Martin Muddu, Anne R. Katahoire, Chris T. Longenecker

**Affiliations:** 1 Department of Internal Medicine, Makerere University, Kampala, Uganda; 2 Infectious Diseases Research Collaboration, Kampala, Uganda; 3 Makerere University Joint AIDS Program, Kampala, Uganda; 4 Division of Adult Cardiology, Uganda Heart Institute, Kampala, Uganda; 5 Uganda Initiative for Integrated Management of Non-Communicable Diseases, Kampala, Uganda; 6 Department of General Internal Medicine, Yale School of Medicine, New Haven, United States of America; 7 Department of Biostatistics and Center for Methods on Implementation and Prevention Science (CMIPS), Yale School of Public Health, New Haven, United States of America; 8 Child Health and Development Centre, Makerere University, Kampala, Uganda; 9 Division of Cardiology, Department of Global Health, University of Washington, Seattle, United States of America

**Keywords:** HIV, hypertension, implementation, integrated care, people living with HIV

## Abstract

**Background:**

The World Health Organization (WHO) recommends integrating hypertension and human immunodeficiency virus (HIV) care; however, evidence for implementing integrated care in primary healthcare (PHC) HIV clinics remains limited.

**Aim:**

To assess the feasibility and acceptability of a pilot model for integrating hypertension care into HIV services and to describe the hypertension care cascade among people living with HIV (PLHIV) and hypertension.

**Setting:**

Two PHC HIV clinics in Wakiso district, Uganda.

**Methods:**

We conducted a parallel convergent mixed methods study. The pilot intervention included providing blood pressure (BP) cuffs, antihypertensive medications, a treatment algorithm and training healthcare provider (HCP) on hypertension care. Quantitative data were collected from February 2022 to December 2022. Using the consolidated framework for implementation research, we conducted interviews with HCPs (n = 12) and PLHIV with hypertension (n = 8) to explore implementation determinants. We performed descriptive analysis for hypertension care cascades. Qualitative data identified barriers and facilitators to integrating HIV and hypertension care.

**Results:**

Of 3802 PLHIV in care, 3502 (92%) were screened for hypertension. Among these, 290 (8.3%) had a chart diagnosis of hypertension, 282 (97.2%) were treated and 128 (50.2%) achieved BP control. Key facilitators included access to medications, BP monitors and improved provider knowledge on management of BP among PLHIV. Barriers included unsynchronised clinic visits and increased provider workload.

**Conclusion:**

Integrating hypertension and HIV services in Ugandan HIV clinics is feasible and acceptable. Availability of resources (BP medications and monitors) and trained personnel facilitates integration of these services.

**Contribution:**

This pilot study provides evidence that integrating hypertension care into existing PHC HIV in Uganda and other similar settings is both feasible and acceptable but may necessitate additional human resources for health.

## Introduction

Sub-saharan Africa region bears the world’s heaviest human immunodeficiency virus (HIV) burden, yet the region has made remarkable progress in combating the epidemic over the past four decades. This success of the HIV programme has been largely attributed to the widespread scale-up of antiretroviral therapy (ART) for all people living with HIV (PLHIV) at no cost, the implementation of the ‘test and treat’ strategy and the decentralisation of ART care responsibilities to non-physician healthcare providers (HCPs). As a result, HIV and/or acquired immunodeficiency syndrome (AIDS)-related mortality has decreased significantly, with a reduction of over two-thirds (69%) between 2004 and 2022.^1^ These efforts have increased life expectancy among PLHIV, leading to an ageing population increasingly at greater risk of non-communicable diseases (NCDs), such as cardiovascular diseases (CVD), cancer and diabetes mellitus. People living with HIV are particularly vulnerable to CVD because of chronic HIV-related chronic inflammation, side effects of some ART and a high prevalence of traditional cardiovascular risk factors.^2^

In Uganda, an estimated 20% – 30% of PLHIV have hypertension, the leading traditional risk factor for CVD in low- and middle-income countries (LMICs).^3,4,5^ For instance, the 2021 Global Burden of Disease study estimates that hypertension in Uganda accounts for nearly five times more disability-adjusted life years than high low density lipid cholesterol.^6^ The high prevalence of hypertension and its substantial contribution to morbidity and mortality underscore the urgent need to integrate hypertension management into HIV programmes at all levels. This model involves delivering both HIV and hypertension care to PLHIV at the same facility, during the same visit and by the same HCPs. Although widely recommended, its implementation remains limited in scope in Uganda, often confined to centres of excellence and tertiary HIV clinics. Several researchers have explored innovative approaches to integrating HIV and hypertension care in Uganda, yielding promising results.^7,8,9,10^ For instance, our team implemented the WHO technical package for cardiovascular diseases management at a tertiary urban HIV clinic and achieved improved hypertension care cascade metrics without compromising HIV outcomes.^7^ However, it remains unclear whether a similar or adapted integration model can be effectively implemented in primary healthcare (PHC) HIV clinics, where HIV care is predominantly provided by non-doctor HCPs and with limited resources.^11^

The PULESA Uganda (Strengthening the Blood Pressure Care and Treatment Cascade for Ugandans Living with HIV – Implementation Strategies to SAve Lives) trial aims to improve blood pressure (BP) care metrics by developing and testing contextually adaptable implementation strategies for integrating HIV-hypertension care in primary care HIV clinics in urban and peri-urban areas in Uganda.^12^ Drawing from the findings of the PULESA Uganda formative research and existing literature on facilitators and barriers to HIV-hypertension care integration in PHC settings,^13,14^ we collaborated with the stakeholders – including HCPs at pilot clinics, PLHIV and hypertension, policymakers, senior disease control programme managers at Wakiso and Kampala districts and the Uganda Ministry of Health – to design and pilot a contextually acceptable integration model. The collaborative process utilised human-centred design (HCD) methodology through three initial phases: (1) inspiration; (2) ideation; and (3) prototyping, followed by pilot testing for acceptability and feasibility. A fourth HCD meeting focused on refining the implementation components. Insights from the pilot study informed iterative development of the multi-component implementation strategy, which is now being evaluated in a cluster-randomised trial involving 16 HIV clinics in Wakiso and Kampala districts for integrating HIV and hypertension.^12,15^ In this article, we present findings on the acceptability and feasibility of implementing this multi-component strategy for integrating HIV-hypertension care in primary care HIV clinics in Uganda based on the consolidated framework implementation research (CFIR).^16^ In addition, we describe the hypertension care cascade among PLHIV with hypertension.

## Research methods and design

### Study design

We conducted a parallel convergent mixed methods study where quantitative and qualitative data were collected and analysed concurrently with equal priority. This pilot implementation study was part of an HCD process that developed strategies for integrating hypertension care into HIV clinics in Uganda. The study occurred from February 2022 to December 2022 in two purposively selected facilities: one public clinic and one private not-for-profit (PNFP) clinic, representing different healthcare delivery models. Quantitative measures focused on hypertension screening, treatment and control rates among PLHIV, while qualitative methods explored implementation barriers and facilitators through interviews with HCPs and patients. Data integration occurred during interpretation to provide comprehensive insights into the implementation process and outcomes.

### Study setting

This study was conducted at two PHC HIV clinics in Wakiso district, Uganda: Clinic A, a public health facility serving approximately 1200 PLHIV, and Clinic B, a PNFP hospital serving over 2000 PLHIV. Both clinics provide comprehensive HIV care, including HIV testing services, treatment and management of HIV-related comorbidities. A multidisciplinary team comprising clinical officers, nurses, HIV counsellors, laboratory technicians and pharmacy technicians delivers these services.

The clinics were purposively selected from 10 HIV clinics that participated in a preceding mixed methods formative study.^17^ Findings from the formative study highlighted differences in HIV-hypertension care integration at the two clinics.^17^ At Clinic A, integration was minimal to non-existent, hypertension screening was not routine and comorbid cases were either managed at the outpatient department or externally. In contrast, Clinic B had partial integration offering BP measurements to some PLHIVs although documentation of these services in the health information tools was inadequate. While PLHIV with hypertension received prescriptions, antihypertensive medications were only available for purchase through the central hospital pharmacy or other private (external) pharmacies, leading to inconsistent adherence.^17^

### Pilot intervention

**FIGURE 1:**
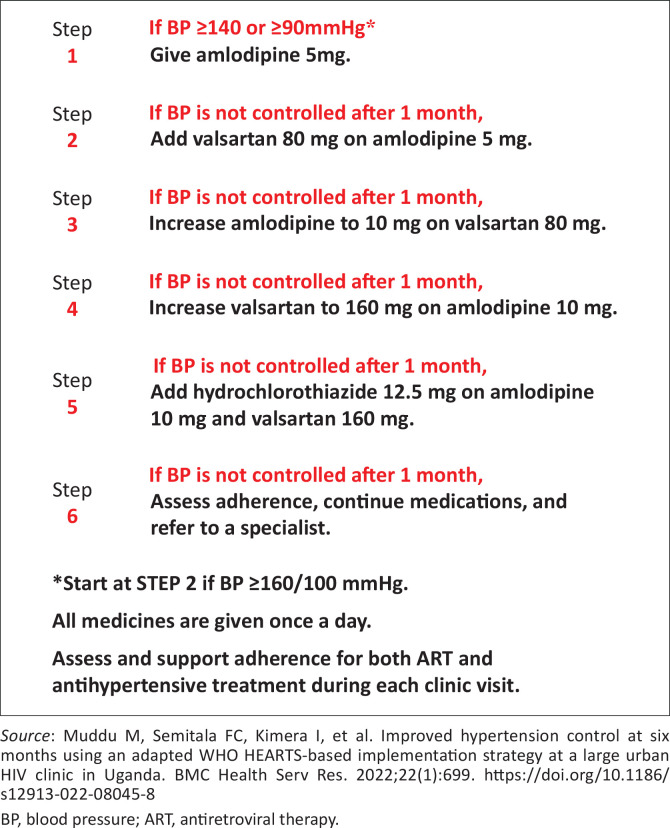
Blood pressure treatment algorithm.

Our intervention was co-designed with key stakeholders to integrate hypertension care into existing HIV service delivery. The intervention package included three main components: (1) essential equipment; (2) medication access; and (3) provider capacity building. We supplied each clinic with at least three automated BP measurement devices (*Omron M1 basic)* and a standardised hypertension treatment algorithm (Figure 1) adapted from national guidelines.^15^ Patients diagnosed with hypertension received hand-held BP monitoring cards to track measurements between visits and facilitate medication titration during clinic appointments. We procured guideline-recommended antihypertensive medications (amlodipine, valsartan and hydrochlorothiazide) through the Novartis Access programme^16,17^ and established distribution systems within the clinics’ existing pharmacy infrastructure.

Our training approach consisted of an initial 2-day comprehensive training (2 h – 3 h per session) for the entire clinic team, covering BP measurement technique, hypertension diagnosis criteria and medication management using the standardised treatment algorithm (Figure 1). Under workflow integration support, the study team worked with clinic staff to incorporate BP screening into triage processes and establish hypertension treatment within existing HIV care pathways. Follow-up support included: a 2-h refresher training at each site 1-month post-implementation; two virtual case discussions via Zoom during the first 2 months and ongoing support supervision and one-on-one coaching by the study doctor and nurse throughout the 9-month implementation period.

We implemented a fully integrated care model where the same clinician provided both HIV and hypertension care during each visit; documentation was integrated into existing HIV health information systems (care cards and dispensing logs), and follow-up appointments were synchronised for both conditions. Screening was led by a peer champion (a PLHIV with hypertension), while treatment efforts were overseen by a designated clinical focal person, typically a clinical officer or nurse. The focal person conducted a routine review of documentation, treatment protocols, resource availability and BP indicators at the clinic level. This approach aimed to minimise additional workload for providers while maximising patient convenience through synchronised care.

### Participant selection

For the quantitative component, we collected routine clinical data from PLHIV aged 18 years and above who received integrated HIV and hypertension care at the pilot clinics. In accordance with Uganda national guidelines, participants were classified as having hypertension if they had at least two BP readings ≥ 140/90 mmHg from separate clinic visits a week apart or were on antihypertensive treatment.^18^ We did not predetermine a sample size; instead we included all eligible participants who sought care at the clinics in the analysis.

For the qualitative component, we purposively selected a sub-sample of 12 PLHIV with hypertension (*n* = 6 per clinic) aged 18 years and above, who provided informed consent to participate in in-depth interviews (IDIs). We excluded those with cognitive impairment or using antihypertensive medications for purposes other than treating hypertension (e.g. systolic heart failure). The purposive sampling strategy aimed to ensure representation by sex and by timing of hypertension diagnosis (either prior to or during the intervention rollout).

For the key informant interviews (KIIs), we purposively selected 8 HCPs (*n* = 4 per clinic) from diverse professional cadres who delivered integrated HIV and hypertension care.

### Data collection

#### Quantitative data collection

Two trained research assistants, one at each clinic, extracted data from paper-based medical records on each clinic visit for all target participants. We collected data from all eligible participants who visited the clinics between February 2022 and December 2022. We entered these data in a pre-designed tool developed in REDCap.^19,20^ Data captured included socio-demographic characteristics for all adult PLHIV and detailed clinical characteristics such as viral load suppression status, HIV and hypertension treatment, BP records and comorbidities for all PLHIV diagnosed with hypertension. The study data manager conducted periodic data quality checks to ensure that all metrics were correctly entered, and also quarterly comparisons between facility electronic medical records and study medical records were made to ensure that information from all PLHIV seeking care at these clinics was captured. The data were exported to Stata version 15 (Stata Corp, Texas, United States) for analysis.

#### Qualitative data collection

Two months after the pilot intervention rollout (April 2022), we conducted qualitative interviews at both clinics. Trained research assistants (J.S., male and C.K., female) carried out the interviews with PLHIV who had hypertension. Interviews were primarily conducted in Luganda, the predominant language in central Uganda. In addition, F.A., a female behavioural social scientist conducted KIIs with HCPs. Key informant interviews were conducted in English. All interviews lasted for 45 min – 60 min and were conducted in a purposively identified private rooms or spaces within the HIV clinics to ensure confidentiality and create a conducive environment for data collection. All interviews were anonymised and transcribed by a third-party service, with subsequent verification for accuracy. The interview guides incorporated questions from the CFIR to assess patients’ acceptability of the intervention.

### Data analysis

#### Quantitative data

We performed univariate analyses to summarise the socio-demographic and other characteristics of the cohort. Continuous variables were described using means and standard deviations (s.d.) or medians and interquartile ranges (IQRs) depending on normality, while categorical variables were summarised as frequencies and percentages. Data were stratified by clinic type (Public-Clinic A and PNFP-Clinic B). Baseline characteristics were compared between the two subgroups using the Chi-square (χ^2^) if counts were ≥ 5 or Fisher exact tests (if counts were < 5) for categorical variables. For continuous variables, *t*-test was employed if the data were normally distributed while Mann-Whitney U test was employed for non-normally distributed data. We conducted descriptive analyses to determine frequencies and percentages of PLHIV at each predefined cascade step relative to the preceding step. Data analyses were performed using Stata 15 (Stata Corp, Texas, US).

#### Definitions of hypertension cascades

We have previously described the hypertension care cascade as follows^11^:

- **Screened:** The number of PLHIV with at least one documented BP measurement divided by the number of PLHIV aged 18 years and above seen at the clinic.
- **Hypertension:** The number of PLHIV with at least two BP readings ≥ 140/90 mmHg on two separate clinic visits (at least a week apart) or those receiving antihypertensive medications.
- **Hypertension diagnosed:** The number of PLHIV with at least two BP readings ≥ 140/90 mmHg on two separate clinic visits at least a week apart or are receiving antihypertensive medications, who have been accurately diagnosed by the clinic staff, divided by the number of PLHIV screened.
- **Treated:** The number of PLHIV with hypertension who have a current prescription for antihypertensive treatment, divided by the total number of PLHIV with hypertension:
- **Monitored:** The number of PLHIV with hypertension who received BP measurements at subsequent clinic visits, divided by those who attended the visit.
- **Controlled:** The number of PLHIV with BP < 140/90 mmHg on the last available clinic visit, divided by the number of PLHIV with hypertension.

#### Qualitative data

Interviews were audio-recorded, transcribed verbatim and translated into English when necessary. Data were analysed using qualitative research standards and reporting International’s NVivo (version 20). Our analysis was anchored in the CFIR.^16^ Three researchers (F.A., C.K. and J.S.) employed a hybrid analytical approach, initially conducting inductive coding following Braun and Clarke’s reflexive thematic analysis methodology.^21^ As analysis progressed, the research team identified relevant CFIR constructs that provided analytical structure for understanding the implementation of integrated HIV and hypertension care. We systematically mapped emergent themes onto corresponding CFIR domains and constructs, as detailed in Table 5. This mapping process illuminated key factors that either supported or hindered the implementation of the integrated care model.

To ensure consistency, the coding team triple-coded sample transcripts to develop a preliminary codebook, which was refined through regular team meetings. After establishing the final coding scheme, researchers independently coded the remaining transcripts. For this analysis, we defined acceptability as stakeholders’ perception that the intervention components were meaningful and aligned with their care preferences and needs. We defined feasibility as stakeholders’ perspectives on how well the integrated care model functioned within existing clinical workflows and resource constraints. Our focus was identifying contextual factors influencing intervention uptake, delivery and perceived value to patients and HCPs.

### Trustworthiness of qualitative data

To ensure trustworthiness, we employed multiple methods to capture perspectives from different participant categories. Transferability was supported through comprehensive documentation, while credibility was enhanced by maintaining detailed field notes, an audit trail and conducting member checking during a stakeholder dissemination meeting.^22^ Feedback from participants informed the refinement of intervention components for the subsequent cluster-randomised trial.

### Data triangulation and integration

Each dataset (qualitative and quantitative) was analysed separately before being integrated to form a comprehensive interpretation – an approach known as mixed methods integration. This strategy allowed us to understand not only how HCPs delivered the intervention but also how patients experienced it. By triangulating data – from quantitative metrics and qualitative insights – we enhanced the validity and credibility of our findings, providing a richer, multifaceted understanding of the intervention in real-world contexts.

### Ethical considerations

Ethical approval to conduct this study was obtained from the Makerere University School of Medicine Research and Ethics Committee (Mak-SOMREC-2021-58) and the Uganda National Council for Science and Technology (No. SS808ES). Administrative clearance was obtained from the Uganda Ministry of Health and the Wakiso District Local Government. In addition, a waiver of consent was granted to collect routine clinical data from PLHIV attending the clinics. Participants who took part in the interviews provided written informed consent before the interviews were conducted.

## Results

### Socio-demographic characteristics

Between February 2022 and December 2022, data were collected from 3802 PLHIV who attended the two clinics. The mean age of participants was 37.1 years (s.d. 10.8), and 68.9% were female (Table 1). Of 3802 PLHIV in care, 3502 (92.0%) were screened for hypertension. Of these, 290 (8.3%) had a chart diagnosis of hypertension, the majority of whom were female (*n* = 190, 65.5%) with a mean age of 47.2 years (s.d. = 11.1). Participants from Clinic A were relatively younger than those in Clinic B ([mean: 44.8, s.d. = 12.5] vs. [mean: 48.3, s.d. = 10.2], *p* ≤ 0.001). More than half of the participants were either overweight or obese (54.1% at Clinic A and 55.5% at Clinic B). In addition, 7% of the participants, all from Clinic B, were receiving HIV care through community-differentiated service delivery (DSD) models (Table 2).

**TABLE 1:** Demographic characteristics of people living with HIV receiving human immunodeficiency virus care at two human immunodeficiency virus clinics in Wakiso, Uganda (*N* = 3802).

Variable	Overall						Clinic A (*n* = 1459)						Clinic B (*n* = 2344) (*n* = 2343)						*p*
	*n*	%	Mean	± s.d.	Median	IQR	*n*	%	Mean	± s.d.	Median	IQR	*n*	%	Mean	± s.d.	Median	IQR	
Age (years)	-	-	37.1	10.8	-	-	-	-	33.8	9.8	-	-	-	-	39.2	10.9	-	-	< 0.001*
Age (years)	-	-	-	-	35	29–43	-	-	-	-	32	27–39	-	-	-	-	38	31–46	< 0.001**
**Gender**																			
Male	1181	31.1	-	-	-	-	447	30.6	-	-	-	-	1609	68.7	-	-	-	-	0.655***
Female	2621	68.9	-	-	-	-	1012	69.4	-	-	-	-	734	31.3	-	-	-	-	-

IQR, interquartile range.

* , *t*-test;

** , Mann-Whitney rank sum test;

*** , Chi-squared test.

**TABLE 2:** Demographic and clinical characteristics for people living with HIV with hypertension at two human immunodeficiency virus (HIV) clinics in Wakiso, Uganda.

Variable	Overall (*N* = 290)						Clinic A (*N* = 89)						Clinic B (*N* = 201)						*p*
	*n*	%	Mean	s.d.	Median	IQR	*n*	%	Mean	s.d.	Median	IQR	*n*	%	Mean	s.d.	Median	IQR	
Age (years)	-	-	47.2	± 11.1	-	-	-	-	44.8	± 12.5	-	-	-	-	48.3	± 10.2	-	-	0.025*
Age (years)	-	-	-	-	46.5	39–55	-	-	-	-	43	36–54	-	-	-	-	48	40–56	0.008**
**Sex**																			
Male	100	34.5	-	-	-	-	34	38.2	-	-	-	-	66	32.8	-	-	-	-	0.375***
Female	190	65.5	-	-	-	-	55	61.8	-	-	-	-	135	67.2	-	-	-	-	-
**Body mass index (*n* = 261)**																			
Underweight (< 18.5 kg)	9	3.5	-	-	-	-	2	3.3	-	-	-	-	7	3.5	-	-	-	-	0.556***
Normal (18.5 kg – < 25 kg)	100	38.3	-	-	-	-	26	42.6	-	-	-	-	74	37.0	-	-	-	-	-
Overweight (25 kg – < 30 kg)	88	33.7	-	-	-	-	16	26.2	-	-	-	-	72	36.0	-	-	-	-	-
Obese (≥ 30 kg)	64	24.5	-	-	-	-	17	27.9	-	-	-	-	47	23.5	-	-	-	-	-
Duration in month (*n* = 242)	-	-	-	-	77	37–109	-	-	-	-	58.5	5–84	-	-	-	-	87	48.5–117.5	0.0001**
ART regimen (*n* = 281)	-	-	-	-	-	-	-	-	-	-	-	-	-	-	-	-	-	-	-
TDF/3TC/DTG	260	92.5	-	-	-	-	85	97.7	-	-	-	-	175	90.2	-	-	-	-	0.002****
TDF/3TC/EFV	2	0.7	-	-	-	-	0		-	-	-	-	2	1.0	-	-	-	-	-
AZT/3TC/DTG	1	0.3	-	-	-	-	1	1.1	-	-	-	-	0		-	-	-	-	-
ABC/3TC/DTG	16	5.7	-	-	-	-	0		-	-	-	-	16	8.3	-	-	-	-	-
ABC/3TC/ATV/r	2	0.7	-	-	-	-	1	1.2	-	-	-	-	1	0.5	-	-	-	-	-
CD4 (*n* = 208)	-	-	-	-	422.5	276.5–588	-	-	-	-	477.5	299–654	-	-	-	-	418.5	274–573	0.2805**
**CD4 categorised (*n* = 213)**				-															
< 50 cells/mm^3^	12	5.6	-	-	-	-	2	5.1	-	-	-	-	10	5.8	-	-	-	-	0.816***
50 cells/mm^3^ – < 100 cells/mm^3^	8	3.8	-	-	-	-	1	2.6	-	-	-	-	7	4.0	-	-	-	-	-
100 cells/mm^3^ – < 200 cells/mm^3^	15	7.0	-		-	-	4	10.3	-	-	-	-	11	6.3	-	-	-	-	-
≥ 200 cells/mm^3^	178	83.6	-	-	-	-	32	82.1	-	-	-	-	146	83.9	-	-	-	-	-
**Viral load/suppression status (*n* = 281)**				-															
Suppressed viral load (< 1000 copies/mL)	254	90.4	-	-	-	-	68	78.2	-	-	-	-	186	95.9	-	-	-	-	< 0.001***
Viral load detectable	1	0.4	-		-	-	0	-	-	-	-	-	1	0.5	-	-	-	-	-
Viral load unavailable	26	9.3	-	-	-	-	19	21.8	-	-	-	-	7	3.6	-	-	-	-	-
**DSD model for HIV and HTN care, *n* = 281**				-															
Facility-based individual management	69	24.6	-	-	-	-	24	27.6	-	-	-	-	45	23.2	-	-	-	-	0.012****
Fast track drug refill	174	61.9	-	-	-	-	55	63.2	-	-	-	-	119	61.3	-	-	-	-	-
Facility-based groups	18	6.4	-	-	-	-	8	9.2	-	-	-	-	10	5.2	-	-	-	-	-
Community drug delivery points	16	5.7	-		-	-	0	-	-	-	-	-	16	8.3	-	-	-	-	-
Client-led ART delivery	4	1.4	-		-	-	0	-	-	-	-	-	4	2.1	-	-	-	-	-

s.d., standrad deviation; ART, antiretroviral therapy; HTN, hypertension; DSD, differentiated service delivery; IQR, interquartile range; CD4; cluster of differentiation 4.

* , *t*-test;

** , Mann-Whitney rank sum test;

*** , chi-squared test;

**** , Fisher’s exact test.

### Hypertension care cascade metrics over time

**FIGURE 2:**
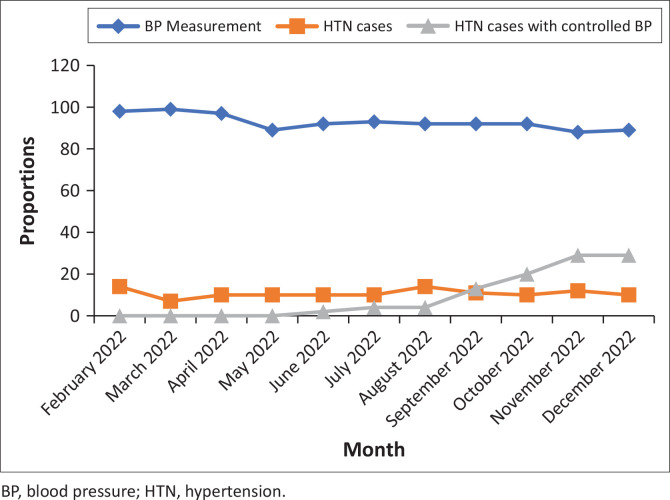
Proportion of adult people living with HIV who received blood pressure measurement, had hypertension diagnosis or had controlled blood pressure by month.

During the first 3 months of the intervention, BP measurements were recorded for over 98%, 99% and 97% of adult PLHIV who attended the clinic in months 0, 1 and 2, respectively (Figure 2). Following intervention rollout, we observed strong performance across various hypertension care cascade metrics. Overall, 92.0% (85.6% at Clinic A and 96.1% at Clinic B, *p* < 0.001) had at least one recorded BP measurement during clinic visits. Of these, 8.3% (7.1% Clinic A and 8.9% Clinic B, *p* = 0.256) were diagnosed with hypertension. Among those diagnosed, 97.0% were initiated on treatment using the study-provided treatment algorithm. At their last clinic visit, 50.0% of PLHIV achieved BP control (< 140/90 mmHg), with control rates of 67.5% at Clinic A and 42.3% at Clinic B, *p* ≤ 0.001 (Table 3).

**TABLE 3:** Hypertension care cascade metrics at two pilot sites.

Variable	Overall (*n* = 290)				Clinic A (*n* = 89)				Clinic B (*n* = 201)				*p*
	*n*	%	Mean	± s.d.	*n*	%	Mean	± s.d.	*n*	%	Mean	± s.d.	
BP measurement at least once	3502	92	-		1250	85.7	-	-	2252	96.1	-	-	< 0.001*
Hypertension diagnosis	290	8.3	-		89	7.1	-	-	201	8.9	-	-	0.063*
Mean baseline SBP	-	-	153	± 27.4	-	-	161.9	± 26.5	-	-	149.3	± 26.9	< 0.001**
Mean baseline DBP	-	-	96	± 18.1	-	-	100.8	± 16.7	-	-	93.7	± 18.3	0.002***
Treated using study algorithm	282	97.2	-	-	88	98.9	-	-	194	96.5	-	-	0.256*
Monitored	255	86.2	-	-	80	87	-	-	175	85.8	-	-	0.787*
Controlled	128	50.2	-	-	54	67.5	-	-	74	42.3	-	-	< 0.001*
Prior hypertension diagnosis	67	23.1	-	-	6	6.7	-	-	61	30.4	-	-	< 0.001*
Prior hypertension diagnosis	81	21.4	-	-	7	5.5	-	-	74	29.6	-	-	< 0.001*
Prior use of hypertension medication	61	16.1	-	-	5	3.9	-	-	56	22.4	-	-	< 0.001*

Note: *p*-value in bold denoted as < 0.001.

s.d., standrad deviation; BP, blood pressure; SBP, systolic blood pressure; DBP, diastolic blood pressure.

* , Chi-squared test;

** , *t*-test;

*** , Mann-Whitney rank sum test.

### Qualitative findings

#### Participant characteristics

Eight HCPs participated in the KIIs. Of these, seven (87.5%) were females with median age was 31 (IQR: 28–44). Out of the 12 PLHIV with hypertension who were interviewed, 6 (50%) were males, and the median age was 49 (IQR: 37–60) (Table 4).

**TABLE 4:** Characteristics of participants who participated in the qualitative interviews.

Variable	Healthcare providers (*n* = 8)				PLHIV with hypertension (*n* = 12)			
	*n*	%	Median	IQR	*n*	%	Median	IQR
Age (years)	-	-	31	28–44	-	-	49	37–60
**Sex**								
Male	1	12.5	-	-	6	50	-	-
Female	7	87.5	-	-	6	50	-	-
**Cadre ship**								
Medical doctor	0	0	-	-	-	-	-	-
Nurse	4	50	-	-	-	-	-	-
Clinical officer	3	37.5	-	-	-	-	-	-
Pharmacy technician	1	**12.**5	-	-	-	-	-	-

Note: Data expressed as median (interquartile range) for continuous variables and number (%) for categorical variables.

IQR, interquartile range; PLHIV, people living with HIV.

The integration of HIV and hypertension care within a unified service delivery model, as assessed through the CFIR framework, uncovered a range of factors that shaped both the perceived facilitators *(denoted with ‘+’)* and barriers *(denoted with ‘−’)* of this innovation. This study examined the findings through the lens of key CFIR constructs, including innovation relative advantage, innovation adaptability, innovation design, inner setting and access to knowledge and information (Table 5).

**TABLE 5:** Mapping barriers to and facilitators of integrated human immunodeficiency virus (HIV) – Hypertension care using the consolidated framework implementation research framework.

CFIR domain	CFIR construct	Barriers/facilitators	Description
Intervention characteristic	Relative advantage	Facilitator	Improved BP screening (92% of adult PLHIV were screened for hypertension) Improved hypertension diagnosis (> 8% of PLHIV diagnosed with hypertension) Reduced fragmentation of care Provided a one-stop shop for HIV and hypertension care Reduced multiple clinic visits and transportation costs.
	Innovation adaptability	Facilitator	Hypertension care aligned with HIV chronic care model Changes within the clinic workflow to incorporate hypertension care.
	Innovation design	Facilitator	Client-centred care Hypertension care fitted well into the HIV care with minimal disruption to the clinic workflow Eliminated the need for multiple clinic visits.
Inner setting	Information and Technology infrastructure	Barrier	Electronic medical records systems not well suited for hypertension metrics. Unavailability of physical registers for longitudinal tracking of hypertension care.
	Availability of resources or materials	Facilitator	Improved BP screening (92% of adult PLHIV were screened for hypertension) Improved hypertension diagnosis (> 8% of PLHIV diagnosed with hypertension) Improved access to hypertension treatment (97% of PLHIV diagnosed with hypertension were treated) Improved BP control (half of the clients achieved BP control) Minimised clinic and pharmacy visits Reduced cost of buying antihypertensive medications
	Structural characteristics	Facilitator	Improved clinic efficiency Allowed task sharing and shifting.
		Barrier	Increased HCP-client interaction time Increased workload because of BP screening and documentation and counselling and prescription refills Increased waiting time for PLHIV.
	Access to information	Facilitator	Tailored training improved HCP’s confidence to provide hypertension care Improve client’s self-efficacy and management of hypertension Simplified treatment algorithm improved prescription of hypertension meds.
	Innovation complexity	Barrier	Initial disruption of HIV DSD models for clients with uncontrolled BPs. Multiple visits for clients with uncontrolled BPs. Misalignment of HIV and hypertension medications refill visits. Increased HCP-client interaction.

HIV, human immunodeficiency virus; DSD, differentiated service delivery; HCP, healthcare providers; PLHIV, people living with HIV; CFIR, consolidated framework implementation research.

### Acceptability

#### Consolidated framework implementation research innovation relative advantage

**Integrated human immunodeficiency virus – Hypertension care preferred to fragmented human immunodeficiency virus and hypertension care:** Both HCPs and PLHIV perceived integrated HIV-hypertension care as superior to the traditional fragmented approach. Healthcare providers, in particular, emphasised the advantages, highlighting increased opportunities for hypertension screening and diagnosis among PLHIV. This improvement was largely attributed to the availability of diagnostic equipment, which enhanced the effectiveness of hypertension detection. The following excerpts from a HCP and a clinical officer illustrate the significant impact screening has had within the integrated HIV-hypertension care:

‘The changes we have made are about taking BP because we have not been doing it [*BP measurement*]. Yes, we have added hypertension to our care.’ (Nurse, Female 27, Clinic A)

‘So, the study [*PULESA Uganda*] has helped us to enrol more [*PLHIV with hypertension*] so in the clinical rooms the clinicians are now taking on the technical role of giving the hypertensive medications because sometimes we would say let this one go to the physician but this time round at least they know now they have to prescribe them the medications are there.’ (Clinical officer, Female 45, Clinic B)

Patients greatly valued receiving both HIV and hypertension care on the same day, as it saved transportation costs and time. They also appreciated the opportunity to learn about their hypertension status during the integrated visit, including access to screening and confirmed diagnoses, a service previously unavailable at their clinics:

‘The first benefit is that I am still alive. Secondly, I would not have known that I am living with hypertension, but when I was screened, I got to know my status, and I am now stronger.’ (PLHIV, Male 38, Clinic A)

### Feasibility

#### Consolidated framework implementation research innovation adaptability

Healthcare providers noted that integrating the intervention to the HIV clinic setting was straightforward, as hypertension care fit will well within the chronic disease management model already used for HIV. At Clinic A, one provider emphasised the importance of establishing a functional triage area to ensure effective screened for all PLHIV attending the HIV clinic:

‘We had to improve the triage; we added a triage table, which wasn’t there before, to allow [*BP*] measurement to take place at this point. As HIV clinic staff, we are supported by peer counsellors, although they were not previously measuring [*BP*] consistently. … With this project [*PULESA Uganda*], we have created a fixed table at the triage where every PLHIV has their weight and MUAC [mid upper arm circumference] taken and their BP added.’ (Nurse, Female 30, Clinic A)

### Facilitators (+)

#### Consolidated framework implementation research innovation design (+)

**Integration of hypertension into human immunodeficiency virus care:** Healthcare providers and PLHIV universally acknowledged the value of integrated HIV-hypertension care as a good model, enabling PLHIV to receive care for both conditions in a single location – a ‘one-stop shop’, eliminating the need for multiple clinic visits. Healthcare providers, in particular, noted that PLHIV would greatly benefit from receiving integrated HIV-hypertension care:

‘Yes, it [*Integrated HIV-hypertension model*] is acceptable because there have been so many studies and I feel that they are well embraced with it because now you find that the BP is well taken. So, with me am thinking your intervention is well embraced because it is something part of them [*healthcare provider*] and now it is a good reminder and a good practice.’ (Clinical officer, Female 45, Clinic B)

Patients agreed that the integrated HIV-hypertension care model was acceptable and aligned well with the existing HIV programme structure. People living with HIV noted that the model met their needs by providing coordinated care for both conditions, effectively addressing previous challenges such as the high cost of antihypertensive medications:

‘If both conditions [*HIV and hypertension*] are handled jointly and treatment is received at once without moving from here to there, it gives us great relief.’ (PLHIV, Female 30, Clinic B)

In addition, PLHIV noted that receiving integrated HIV-hypertension care at the same location and from the same HCP eliminated the need for multiple clinic visits. This reduced the burdens of frequent trips and helped them save on transportation costs. One PLHIV, as illustrated in the following excerpt, describes how the integrated care model effectively addressed their needs:

‘It’s economical in such a way that I come once and I get both services. Meaning I save on my transport. Where I come from, I use twenty thousand [*≈5.4 USD*] Uganda shillings to and from. It’s better for me that way than coming for HIV services today. Again, you come back the following day for hypertension care. It saves on my transport. In simple terms, it’s a good deal.’ (PLHIV, female 50, Clinic A)

### Barriers (−)

#### Consolidated framework implementation research inner setting

**Consolidated framework implementation research information technology infrastructure (−):** Healthcare providers at Clinic B reported that the integration of HIV-hypertension care improved clinic workflows and strengthened documentation for tracking PLHIV with hypertension. However, they highlighted challenges with the existing electronic medical records system, noting that while it effectively captured HIV-related data, it lacked the infrastructure to record hypertension indicators, limiting their ability to track PLHIV with hypertension over time:

‘Another issue with the EMR system it also doesn’t have the provision for hypertension indicators but the system has a provision where you can include a record on other medications so that’s where we put antihypertensive medications …’ ‘Another issue with the EMR system is that it doesn’t have space, but the system has where you put other medications, so that’s where we put antihypertensive medications. So, it has been added to the documentation, but on clerkship and treatment, only a little apart from the documentation. The only thing is the flow because now we have a few more PLHIV to prescribe for, mainly those for hypertension, but it still fits in the programme; it still fits there, really.’ (Clinical officer, Male 29, Clinic B)

### Facilitator (+)

#### Available resources or materials and equipment (+)

**Availability of blood pressure screening devices:** The availability of BP monitoring devices was recognised as critical to integrating HIV-hypertension care, addressing previous gaps and enabling effective diagnosis and monitoring of hypertension among PLHIV. Access to essential resources, such as antihypertensive medications, was also seen as a key enabler of integration. As shown in Figure 2 and Table 3, BP measurement rates increased after the pilot intervention, with nearly all PLHIV receiving BP assessments during clinic visits. The intervention also enhanced the identification and monitoring of PLHIV with hypertension.

**Access to antihypertensive medication at no cost:** People living with HIV valued the provision of free antihypertensive medications within the integrated HIV-hypertension care model, describing it as significant financial relief that eased concerns about managing hypertension. Combining the provision of medications for both conditions also minimised the need for frequent clinic visits, reducing travel-related inconveniences and associated transportation costs:

‘[Integration of HIV-hypertension care] helped us [PLHIV] so much from worrying about hypertension, which may even intensify the illness. Here, even when you get so worried, you already have medication to control your BP, and getting both treatments at once saves us time, transport costs, and other inconveniences. With this new arrangement, we hit two birds with one stone.’ (PLHIV, female 44, Clinic B)

The availability of antihypertensive medications and a simplified prescribing algorithm facilitated the integration of hypertension care into the HIV programme. This enabled HCPs to offer streamlined care, including adherence counselling, self-management education and medication refills, alongside existing HIV services, effectively addressing a critical resource gap:

‘It [*availability of anti-hypertensives*] has improved our service generally, especially for PLHIV with hypertension. Previously, we would advise PLHIV with hypertension to go and buy the [hypertension] medication, we would write a prescription, and then they [*PLHIV with hypertension*] would tell us that for two days, we didn’t take it because we did not have money. Other clients [*PLHIV with hypertension*] would say they didn’t care if they died because they didn’t have money for the medications and they were not free. It has helped us manage the disease since the patient is now aware of it. This has greatly helped us improve our delivery service, and the patients are also pleased.’ (Clinical officer, Female 45, Clinic B)

‘They have helped us, the HIV patients so much, from worrying about hypertension which may even intensify the illness. Here, even when you get so worried you already have medication to control your BP, and getting both treatments at ago saves us time, transport costs and other inconveniences. With this new arrangement, we hit two birds with one stone. What I have liked most is being aware of the state of my BP, secondly is getting medication and advice about managing hypertension.’ (PLHIV, Female 44, Clinic B)

#### Structural characteristics – Work infrastructure (±)

Healthcare providers explained that implementing integrated HIV and hypertension care initially required additional staff because of the increased workload from screening and managing higher patient volumes. They emphasised that many clinic activities could be conducted more efficiently with greater staff support. Despite these staffing challenges, HCP successfully delivered integrated hypertension care. However, they noted that increased waiting times posed an initial barrier, as PLHIVs had to wait longer than they previously did, necessitating adjustments to manage effective flow of the services:

‘There is a great improvement because we were not measuring BP. People [*PLHIV with hypertension*] were going away with their hypertension, and now we can screen them […] Only that, that thing of taking the BP for every PLHIV, if we did not have the interns. It will be difficult. We have nothing to do. We will be trying.’ (Clinical officer, Female 27, Clinic A)

Healthcare providers observed that synchronisation of HIV and hypertension clinic visits reduced the frequent patient visits, enhancing convenience and continuity of care. However, work infrastructure varied across clinics. In Clinic A, hypertension care was delivered by a multidisciplinary team, where peers (PLHIV support staff) assisted BP measurement, while nurses handle treatment prescriptions. In contrast, in Clinic B, integrated care was provided solely by nurses and clinical officers:

‘It [*Integrated HIV-hypertension care*] has improved a lot on our service generally especially for the PLHIV with hypertension, you see previously we would tell the PLHIV you go and buy this [*Medication*] and you write very well the prescriptions and then the patient tells you that the other two days I didn’t take it because I did not have money then, the other patients would tell you I don’t care if I die because now if I don’t have money for the medications because the medications were not free but these ones we are providing are free so, it has equally helped manage the disease that he or she is aware of so it has really helped us a lot to improve on our service delivery and the patients are also very happy.’ (Clinical officer, Female 45, Clinic B)

### Facilitator (+)

#### Consolidated framework implementation research access to knowledge and information (+)

**Training on hypertension care, treatment and management:** Healthcare providers reported a significant improvement in their knowledge and confidence in hypertension care following the study’s training. They praised the training’s comprehensiveness, practical content, particularly the adapted hypertension treatment algorithm, which enhanced screening, diagnosis and treatment.

The training also increased their awareness of hypertension management in PLHIV, providing clear guidance on prescribing, dose titration and managing drug interactions. As a result, they gained greater confidence in delivering integrated care and optimising hypertension management within this population:

‘It’s true before PULESA came in, we had little knowledge about hypertension. What we knew is what we were taught in school because we are entirely focused on HIV. For me, the training has been so helpful. Now I feel comfortable and I prescribe [*anti-hypertensives*] comfortably without any doubt because we were even given information leaflets as backup in case one forgets something, one can always check on these guides.’ (Clinical officer, Female 30, Clinic B)

In addition, HCPs felt that the hypertension treatment algorithm empowered them to confidently prescribe medications and manage patient care; they described using it as a reference point to guide the treatment process:

‘The [*hypertension treatment*] algorithm is very key and has empowered us a lot because it reminds me that in adult learning, you need to have these things that keep reminding you; you cannot cram, but the algorithm is very key because it has awakened the brain.’ (Clinical officer, Female 45, Clinic B)

They were reportedly also able to educate clients more confidently through health education and lifestyle counselling:

‘With lifestyle counselling changes, we have been mentioning these daily because these are the daily things we have been saying, but written or a write-out reminds me that this is what I should do.’ (Clinical officer, Female 30, Clinic A)

### Barrier (−)

#### Consolidated framework implementation research innovation complexity (−)

**Alignment of hypertension care with existing human immunodeficiency virus differentiated service delivery models:** People living with HIV with hypertension identified innovation complexity as a barrier. They explained that their HIV and hypertension appointments were initially unsynchronised, requiring multiple clinic visits for hypertension care. They emphasised the significant financial burden caused by these frequent visits and expressed hope that appointments for HIV and hypertension care would be harmonised in future:

‘It has affected me transport-wise. It would be better if they gave [*me*] both treatments [*HIV and hypertension medications*] for a more extended and equal period. You have to return several days for different treatments as and when any gets finished.’ (PLHIV, Male 60, Clinic B)

‘I request to pick medication for HIV and hypertension at once to save transport money picking at different times. For now, HIV medication is fetched after three months, yet one for hypertension is for one month. It would be better if both were matched to three months. I have to spend money monthly, yet I would only be spending once in three months and get time for my poultry farm.’ (PLHIV, female 54, Clinic B)

Workload-related barriers that clinics have historically experienced when new intervention is introduced. HCPs reflected on the increased time spent on screening, health talks and adherence support for hypertension care:

‘The only thing is that here is too much work load for one person. Yes. In fact, sometimes we forget to document, everyone has a BP taken but you forget to document and you find that person has missed out because you think you’ve not taken.’ (Nurse, Female 30, Clinic A)

‘I wouldn’t say there is an increase in the workload, but for the waiting time, yes, for the PLHIV with hypertensions, it has increased a little bit of their waiting time.’ (Clinical officer, Female 45, Clinic B)

In summary, integrating HIV and hypertension care into a single service delivery model was widely recognised for its benefits, including improved convenience, accessibility and cost savings for both HCPs and patients. However, participants also highlighted key challenges, such as limitations in information technology infrastructure, increased workload and difficulties in synchronising care schedules. Despite these obstacles, the integrated model was largely viewed as a feasible and adaptable approach to addressing the dual healthcare needs of PLHIV who also had hypertension. These findings underscore the model’s potential to enhance care delivery while highlighting areas for further improvement.

## Discussion

This pilot study assessed the acceptability and feasibility of an integrated HIV and hypertension implementation strategy at two PHC HIV clinics in Wakiso district, Uganda. The findings demonstrate that HIV clinics within PHC settings can be effectively leveraged to manage hypertension. Both PLHIV and HCPs found the PULESA Uganda strategies acceptable and feasible for integration in similar contexts. The integrated HIV-hypertension model yielded several benefits: it improved hypertension cascade metrics; established a one-stop shop for HIV and hypertension care, thereby reducing fragmented service delivery; and lowered patient time and transportation costs. Healthcare providers also valued the intervention, noting that it supported person-centred care, strengthened their capacity to manage hypertension and facilitated task-shifting to non-medical doctor cadres. However, despite these positive outcomes, PLHIV and HCPs highlighted areas for improvement to optimise integration. These areas included: aligning HIV and hypertension clinic appointments, adapting DSD models for hypertension management and enhancing monitoring and evaluation systems to support longitudinal tracking of hypertension outcomes.

The PULESA Uganda pilot intervention showed increased performance in BP measurement, diagnosis and control. By end of the study, half of the participants had achieved BP control, with unexpectedly and significantly higher control rates at the public clinic – despite its limited HIV-hypertension integration at baseline and a higher mean baseline BP among the PLHIV. Although the overall BP control rate was slightly lower than the 74% reported in a similar intervention at a tertiary urban HIV clinic in Uganda, key contextual differences may explain this variation.^7^ Unlike the previous study,^7^ this pilot employed an open cohort design, with participants contributing varying durations of follow-up; some experiencing the intervention for a shorter time and others for longer. Despite these differences, the improved clinical outcomes observed in this pilot add to the growing evidence supporting the feasibility and value of integrating hypertension care into HIV services within PHC settings.^7,9^

Preceding formative research and existing literature identified unavailability of antihypertensive medications and limited access to BP devices as key barriers to integrating HIV-hypertension care within HIV clinics.^14,23^ To address these gaps, this pilot leveraged low-cost medications obtained through the Novartis Access programme to strengthen the antihypertensive supply chain. Guided by the CFIR, the study found that improved access to medications was a critical enabler-enhancing HCPs’ motivation to deliver person-centred care and contributing significantly to improved hypertension outcomes. The availability of medications at the clinics not only alleviated the financial burden on the service users but also reduced the need for frequent clinic or pharmacy visits, consistent with findings from both our formative research and other studies.^24,25,26,27^ To effectively scale up the integration of HIV-hypertension care, policymakers should consider providing effective antihypertensive medications available at no cost – like HIV treatment – or at a subsidised cost to those in need. Our ongoing trial will provide comprehensive cost data to inform policy about the investment needed to sustainably deliver integrated care and improve hypertension control at scale.^12^

This pilot implementation strategy addressed low hypertension knowledge among HCPs through a combination of onsite in-person training, virtual sessions via Zoom and individualised coaching.^13,14^ Training materials were tailored specifically for non-physician HCPs – the primary providers of HIV care in Uganda^13^ – and included a simplified treatment algorithm adapted from WHO tools, previously piloted at a large tertiary HIV clinic.^7,28,29^ This approach provided clear guidance on hypertension diagnosis, prescription, titration and treatment optimisation. The targeted training significantly enhanced HCPs’ capacity to deliver hypertension care, enabling effective task shifting and sharing.^13,14^ Tasks such as BP measurement, lifestyle education and adherence counselling were delegated to lay health workers (peer counsellors and expert clients), whose roles in HIV care are well documented in African settings.^30^ Nurses and clinical officers (mid-level healthcare professionals) managed hypertension diagnosis, prescriptions and medication refills. The training was well received, with HCPs reporting increased and improved delivery of health education and counselling. Based on their feedback, the materials were refined for broader roll out of hypertension training in the ongoing trial.

Using the updated CFIR framework,^16^ the study also identified increased workload and extended HCP-patient interaction time as initial barriers to implementation of integrated HIV-hypertension care. In the early phase, HCPs faced additional responsibilities, including BP monitoring, lifestyle counselling and hypertension documentation – tasks not previously part of the routine HIV care. Similar trends have been reported in other studies integrating NCD services into HIV care.^31^ However, this increased burden typically diminishes as HCPs adopt to their expanded roles.^31^ Patients also reported spending more time at the clinic, especially those with uncontrolled BP or new hypertension diagnoses, who required more frequent visits and support. This contrasts with the majority of PLHIV who, because of stable adherence and viral suppression, are eligible for multi-month ART dispensing.^18,32^ These findings informed the adoption of DSD models for our trial, aiming to align HIV and hypertension care within person-preferred frameworks.^12^ The trial tracks HCP-patient interaction times to assess time demands and determine whether additional resources are needed to sustain integrated HIV-hypertension care.^12^

### Study strength and limitations

Our study findings should be interpreted in light of several limitations, although these are mitigated by key aspects of the study design and implementation. Firstly, the study team made multiple visits to the clinics for support supervision, data collection and distribution of antihypertensive medications. While such visits may have influenced HCPs to enhance practices such as BP measurement because of perceived monitoring, they also reflect a realistic model of supportive supervision essential for effective implementation, especially in resource-limited settings. Moreover, improvements in clinical practice were supported by structured training, simplified treatment algorithms and strengthened supply chains, suggesting that observed outcomes were driven by more than just observation bias.

Secondly, the unequal follow-up duration among participants – because of the open cohort design – may have introduced bias in estimating the full impact of the intervention on outcomes such as BP control. However, this approach mirrors real-world clinical settings where patients initiate care at different times and receive care for variable durations. Despite this, the intervention still demonstrated substantial improvements in hypertension metrics, supporting its potential effectiveness even within these constraints.

Thirdly, while qualitative interviews were conducted only 2 months into implementation and lacked follow-up, they provided valuable early insights into acceptability and feasibility. The ongoing trial includes provisions for longitudinal qualitative assessments, which will enable a more comprehensive exploration of contextual factors influencing sustained integration of hypertension care into HIV services over time.

## Conclusion

This pilot implementation study demonstrates that integrating HIV and hypertension services within PHC HIV clinics is feasible and acceptable. Guided by the CFIR framework, we identified critical facilitators such as improved access to antihypertensive medications, availability of BP monitoring devices and strengthened HCP knowledge through tailored training and simplified treatment protocols. These factors contributed to improved hypertension care delivery, task-shifting to non-physician providers and enhanced person-centred care. However, key barriers – including misaligned HIV and hypertension clinic schedules and increased provider workload – were also observed.

To maximise the impact and sustainability of integrated care, we recommend aligning hypertension management with HIV DSD models to streamline visits and reduce client and provider burden. Policymakers should prioritise the availability of affordable or no-cost antihypertensive medications within primary care settings, mirroring the HIV treatment model. In addition, implementation efforts should include structured training, support supervision and routine monitoring of provider-patient interaction time to inform resource needs. These strategies will be essential for scaling integrated HIV-hypertension services and improving long-term outcomes for PLHIV and hypertension.
